# Neurorobotic Models of Neurological Disorders: A Mini Review

**DOI:** 10.3389/fnbot.2021.634045

**Published:** 2021-03-19

**Authors:** Savva Pronin, Liam Wellacott, Jhielson Pimentel, Renan C. Moioli, Patricia A. Vargas

**Affiliations:** ^1^Robotics Laboratory, Edinburgh Centre for Robotics, Heriot-Watt University, Edinburgh, United Kingdom; ^2^College of Medicine and Veterinary Medicine, University of Edinburgh, Edinburgh, United Kingdom; ^3^Bioinformatics Multidisciplinary Environment, Digital Metropolis Institute, Federal University of Rio Grande do Norte, Natal, Brazil

**Keywords:** neurorobotics, robot models, computational models, simulation, pathophysiology, neurophysiology, artificial neural networks

## Abstract

Modeling is widely used in biomedical research to gain insights into pathophysiology and treatment of neurological disorders but existing models, such as animal models and computational models, are limited in generalizability to humans and are restricted in the scope of possible experiments. Robotics offers a potential complementary modeling platform, with advantages such as embodiment and physical environmental interaction yet with easily monitored and adjustable parameters. In this review, we discuss the different types of models used in biomedical research and summarize the existing neurorobotics models of neurological disorders. We detail the pertinent findings of these robot models which would not have been possible through other modeling platforms. We also highlight the existing limitations in a wider uptake of robot models for neurological disorders and suggest future directions for the field.

## 1. Introduction

Modeling is widely used in contemporary biomedical research to gain insights into pathophysiology and treatment of neurological disorders. Models may share physiological, behavioral, or other characteristics with humans yet allow for manipulations that are not possible in human studies, and allow faster data collection. They have contributed to our knowledge in a variety of fields by improving our understanding of pathophysiology, from genetics and molecular biology to systemic neural disturbance and behaviors, and guide the search for therapeutic solutions (Ericsson et al., 2013). However, the use of animal models for predicting the effectiveness of treatment strategies in clinical trials has remained controversial due to (1) their translational failure, which may be explained in part by methodological flaws in animal studies and critical disparities between the animal models and the clinical trials testing treatment strategies (van der Worp et al., 2010), and (2) the guiding principles for ethical use of animals in research, in particular the “Replacement, Reduction, and Refinement” principle (Russell and Burch, 1960). Furthermore, there is increased focus in ascertaining the impact of social interactions on neurological disorders (Kennedy and Adolphs, 2012). The rise of computational modeling and robotics may complement and overcome some of these limitations. This review aims to expand and summarize all published robot models designed to mimic prevalent neurological disorders by detailing their methodologies, experimental paradigms, and translational outcomes. It further aims to contextualize robotics within the various modeling platforms; and act as a reference guide to allow further development of the approach.

### 1.1. Computational Neural Models

Computational neural models can focus on specific parts of the nervous system in isolation, or account for changes to a single neural population in a complex multilevel system, as well as extract highly-detailed data that are difficult to obtain from animal models on causative mechanism, neural dynamics, and treatments (Humphries et al., 2006, 2018). Nevertheless, computational neural models are limited by being a partial implementation of a complete system. For example, fully modeling the human brain is not yet computationally tractable due its high complexity architecture—86 billion neurons running in parallel each with an average of 7,000 connections to other neurons^1^(Richardson, 2017).

Computational models can reveal behaviors that are beyond the scope of non-computational ones. For example when characterizing visual attentional deficits (i.e., neglect) resulting from stroke during viewing a straight line, Mozer et al. (1997)'s computational model showed that neglect was related to the total line length, despite the patient not apprehending the entire line. Additionally, electrophysiological data from animal models, such as monkey single-cell recordings during a visual attention task (e.g., Lanyon and Denham, 2004), can be used to implement computational models. Due to the ability to adjust neural function, computational models are able to explain symptoms of disorders using simple and neurologically plausible principles, such as the prediction error minimization mechanism to explain autism symptoms even if the underlying pathophysiology has not yet been identified (Lawson et al., 2014).

Parkinson's Disease (PD) mechanisms are frequently contextualized by network models, allowing easy translation to computational modeling (Albin et al., 1989; McGregor and Nelson, 2019). A variety of computational models of PD exist mostly focusing on the basal ganglia circuit (Humphries et al., 2018). For example, Frank (2005) designed a neural network model of the basal ganglia during learning and execution of a cognitive—weather prediction task. This model showed that the basal ganglia modulates the execution of actions that are being considered in the frontal cortex, including working memory updating, through phasic changes in dopamine, and that reduced dynamic range of dopamine can explain both PD and dopamine-overdose through a single underlying dysfunction. Dovzhenok and Rubchinsky (2012) and Caligiore et al. (2019) explored PD motor symptoms using a model of basal ganglia-thalamo-cortical loops. Dovzhenok and Rubchinsky (2012) utilized a simple model of a single neuron representing each area, whereas Caligiore et al. (2019) represented the areas with neural populations and introduced multiple interventions representing different dopaminergic dysfunctions. In another experiment, Kumaravelu et al. (2016) used a similar cortex-basal ganglia-thalamus network in the 6-OHDA lesioned rat model of PD to simulate different modes of deep brain stimulation.

### 1.2. Neurorobotic Models

Embodiment of computational neural models in a robot allows for direct sensory-action closed-loop interactions with a real environment as well as with environmental noise, improving the generalizability of experiments to real world scenarios. This can be advantageous for both understanding human cognition (e.g., Chaminade and Cheng, 2009; Di Nuovo et al., 2013) and neurological or psychiatric disorders, particularly because the latter are often characterized by behavior and interaction (e.g., Smith and Gasser, 2005; Asada et al., 2009). For example, nearly all psychiatric and many neurological disorders diagnoses are made clinically, without reliable imaging or biomarker tests (WHO, 1993), thus robotic models are natural candidates to study them by embodying the symptoms as a starting point, in comparison to purely computational models that rely on neuropathological knowledge to construct. One suitable candidate for neurorobotics studies is PD because many of the symptoms seen relate to environmental interactions (Poewe et al., 2017). Robot models can both inform biological data and be hypothesis-generating. Robotics allows for precise implementation of theoretical models and consequential controlled manipulation that is not possible in animal models because of ethical reasons or limited methodology. Robot models increase the replicability of experiments and avoid the issues of subject fatigue allowing for both more and quicker experiments, as well as avoiding the effects of fatigue on performance. Therefore, robot models use resources efficiently and minimize animal studies.

#### 1.2.1. Neurorobotic Models Using Virtual Simulation

Computational models have been implemented into virtual robot simulations, thus bypassing the actual construction of the robot but compromising by having an equally simulated environment. These have included more simplified machines such as simulating a 2D robot leg using a neural system with a hierarchical central pattern generator to model leg weakness as a symptom of stroke to explore rehabilitation strategies (Ichimura and Yamazaki, 2019). Another study simulated a two-link robot arm using a basal ganglia neuron model to examine the relation of neuron potential strength and gradient with symptoms in PD through varying dopamine (Connolly et al., 2000). This model found that heterogeneity of striatal neuron potentials may be responsible for replicated dyskinetic symptoms, such as bradykinesia, rigidity, and tremor.

More complex robots have been also used in this context but with different approaches. Pio-Lopez et al. (2016) investigated the concept of active inference—a mechanism of minimizing variation in perceptual prediction—using a 7-degrees of freedom arm of a PR2 robot. They manipulated visual and proprioceptive noise to investigate prediction errors; one finding suggested that the failure of sensory attenuation would subvert movements, similarly to bradykinesia in PD. Another experiment simulated bipolar-affective disorder (BPAD) and Alzheimer's disease in a NAO robot, to investigate the role of emotion, specifically pleasure, and arousal, in memory (Allan et al., 2015). Researchers used Naïve Bayes Data Mining to train the robot then simulated a RoboCup match penalty kick using the Webots simulator. They found that emotional deregulation after a certain stress threshold in the BPAD model whereas the Alzheimer's disease model, which only memorized events of high emotional value, maintained a relatively high level of emotion throughout the experiment.

In some cases, computational neural models have been proposed to investigate disorders implicitly. For example, by modeling synaptic plasticity such as the wheeled robot of Aguilera et al. (2015). They evolved the model using a genetic algorithm, and controlled it by a network of Kuramoto oscillators with homeostatic plasticity to develop behavioral task preferences mediated by sensorimotor patterns. Similarly, Caligiore et al. (2014) used a simulated iCub robot with a transfer expert reinforcement learning (TERL) neural network to investigate sensorimotor learning during childhood development and how simulated “damage” to the network may have implications for autistic spectrum disorder (ASD).

## 2. Neurorobotics Models of Neurological Disorders

Here we discuss and summarize the existing neurorobotic models of prevalent neurological disorders and the experiments conducted with them (see Table 1 for a summary).

**Table 1:** Summary of neurorobotics models of neurological disorders.

**References**	**Type**	**Disease**	**Class of ANN**	**Robot**	**Experiment**	**Training algorithm**	**External stimuli**	**Internal stimuli**	**Disease simulation**
Lewis and Cañamero (2019)	Article	OCD	–	Elisa-3	Free-roam—Feed, avoid, groom	–	Vision^*^	“Energy,” “Integrity,” “Integument”	Increased parameters
Lewis et al. (2019)	Conference	OCD	As above					As above + “Stress”	Induced “stress” externally
Idei et al. (2018)	Article	Autism	S-CTRNN	NAO	Ball-playing interaction task	Back-propagation through time	Proprioception, Vision	Prediction	Altered sensory variance
Idei et al. (2017)	Conference	Autism	Duplicate of above						
Conti et al. (2016)	Article	Hemispatial neglect	Multi-layer perceptron	iCub	Object manipulation task	Gradient descent with momentum backpropagation	Proprioception	–	Nullified parameters
Yamashita and Tani (2012)	Article	Schizophrenia	CTRNN/ MTRNN	QRIO	Object manipulation task	Back-propagation through time	Proprioception, Vision	Prediction	Addition of different degrees of noise between connections
Yiping et al. (2010)	Conference	PD and HD	Spiking neural network	Lego	Free-roam—wander, avoid obstacles, play	Genetic	Vision^**^	“Safety,” “Boredom”	Reduced parameters

*CTRNN, continuous time recurrent neural network; HD, Huntington's disease; MTRNN, multiple time scale recurrent neural network; OCD, obsessive-compulsive disorder; PD, Parkinson's disease; S-CTRNN, stochastic-continuous time recurrent neural network*.

* *Using an infrared detector*.

** *Using an ultrasonic distance detector*.

### 2.1. Parkinson's Disease (PD) and Huntington's Disease (HD)

Yiping et al. (2010) presented a model imitating PD and HD, another basal ganglia disorder resulting in hyperkinetic movements. The authors simulated dopaminergic neuron death in a two-channel behavior selection model of the basal ganglia, constructed with leaky-integrator artificial neurons, and optimized with a genetic algorithm. This was embedded into the control architecture of a Lego robot. The robot would alternate between wander, avoid, and play behaviors in an open space. The PD robot did not perform any behavior in almost half of the experiment and its behavior sequence separated into short fragments, compared to normal control. The HD robot, instead, had the tendency to execute any of the behaviors even if the respective importance value was small, therefore simulating the behavior patterns of the two diseases in a real-world environment demonstrating the viability of the modeling platform. However, ultimately, the model is an abstraction by substituting behavior selection for the symptoms of hypo/hyperkinesia; this limitation can be overcome by linking the neural controller to musculoskeletal architecture and incorporating further brain regions such as the motor cortex in order to mimic the physical disease symptoms.

### 2.2. Unilateral Spatial Neglect

Unilateral spatial neglect is a disorder of processing and perceiving stimuli, caused not by a loss of sensation but stroke or tumor, on one side of the visual field often contralateral to the damaged cerebral hemisphere, typically the posterior parietal cortex. It is a heterogeneous condition due to the variety of associated lesion locations and may required type-specific rehabilitation as a result. To explore this hypothesis, Conti et al. (2016) embedded an artificial neural network that controlled the spatial attention of an iCub robot. They replicated a human experiment by having the robot perform a spatial exploration task of exploring cubes in four different orientations using proprioceptive stimuli, followed by rehabilitation sessions. They used stochastic gradient descent with momentum to train the neural network, then “pruned” neural links to simulate lesions, and re-applied the training to simulate rehabilitation. Furthermore, they modeled specialization of the right hemisphere by incorporating a plasticity mechanism into some trials of the experiment. Their findings were in line with those in human studies, validating the use of a robot model with environmental interaction in this disorder, but additionally they were able to hypothesize novel plasticity mechanisms of the right hemisphere in spatial specialization. Their model too included a degree of abstraction by simplifying the neural controller to hemispheres rather than specific cortical regions therefore limiting anatomical correlation with humans. Nevertheless, the latter also confers an advantage by allowing study of an isolated function whereas in a complex brain other functions would be affected too by the lesion thus making it difficult to investigate in other models. Future work could also implement additional stimuli such as vision and touch.

### 2.3. Obsessive-Compulsive Disorder (OCD)

Obsessive-compulsive disorder is characterized by repetitive intrusive thoughts resulting in the need to perform certain actions repeatedly. Lewis et al. (2019) conceptualized OCD as a decision-making disorder and designed a robot based on a cybernetic and signal attenuation model within a motivation-based robot controller with an internal signal deficit (faulty interoception). The Elisa-3, an Arduino-based robot, performed a free-roam task requiring decisions to satisfy its needs—energy, integrity, and integument—by utilizing resources using three behaviors—groom (with a damage penalty), feed, and avoid. To simulate OCD, the perceived target values for integument were increased, resulting in increased grooming with many robots “dying” as a result of energy falling to zero whilst grooming and an average worse level of well-being. Interestingly, mildly raised target values improved average well-being.

The authors also further developed the model by implementing “stress”—both internal, related to its needs, and external, due to confinement (Lewis and Cañamero, 2019). Also, rather than changing the target values to simulate OCD, this model would express OCD behaviors resulting from high “stress.” They found a positive feedback loop which maintained high stress levels after the robot was confined, due to a consequential increase in internally generated stress because of not meeting its needs. Additionally, they tried different type of intervention to mimic response prevention therapy with opposite stress response depending on the type of intervention. Similar to Conti et al. (2016)'s model previously, the ability to monitor internal parameters through a robot model led to their findings above. These models are abstract without an artificial neural network but allow investigation of OCD, where the pathophysiology has not been ascertained; future work can incorporate an artificial neural network and simulate pharmacological treatments to investigate the underlying neural correlates.

### 2.4. Schizophrenia

Schizophrenia is a psychiatric disorder characterized by symptoms of hallucination, delusion, and disorganized thought. Yamashita and Tani (2012) created a model using a SONY QRIO humanoid robot controlled by a hierarchical continuous time recurrent neural network with a multiple time scale recurrent neural network component. For more models of Schizophrenia, please check the survey paper of Lanillos et al. (2020). The robot performed a manipulation task following rules associated with object position using proprioception and vision, which generated forward predictions of the two stimuli, learned through a back-propagation through time algorithm. Schizophrenia was simulated through degrees of functional disconnection in the hierarchical network by adding random noise. Mild disconnection led to a spontaneous intermittent increase of aberrant prediction errors which affected the goal-orientation of the robot, whereas severe disconnection resulted in cataplectic and stereotypical behaviors, similarly to what is seen in patients. Translationally, they hypothesized that prediction error signals may be indistinguishable in a patient with mild schizophrenia from those generated from real stimuli and resulting in a prodromal delusional mood, demonstrating an advantage of neurorobotics modeling.

This model manages to explore a non-anatomically localized network-wide neural process in a real-world environment, difficult to achieve using other models; further work could take advantage of internal monitoring to aim to concurrently simulate other schizophrenia symptoms such as delusions and hallucinations to assess whether a similar mechanism is responsible. Other models also use neural networks but focus on specific symptoms, like deficit in the recognition (Cohen and Billard, 2016).

### 2.5. Autism Spectrum Disorder (ASD)

ASD encompasses a range of neurodevelopment disorders affecting mostly social interaction and presenting restricted, repetitive patterns of behavior. Focusing on behavioral inflexibility, Idei et al. (2017, 2018) used a NAO robot to investigate the effects of sensory precision on adaptive behavior. They implemented a stochastic-continuous time recurrent neural network with parametric bias, which is a more complex architecture compared to the first approaches (Cohen, 1994). For more models of ASD, please check the survey paper of Lanillos et al. (2020). The robot participated in an adaptive interaction ball-playing task using vision and proprioception with a human experimenter. It learned four different behaviors using the back-propagation through time algorithm. The level of sensory precision was increased or decreased by changing estimated sensory variance and prediction error signal level was analyzed. Both changes resulted in similar inflexible behavior patterns, such as repetition and freezing seen in patients with ASD, but due to different processes at the network level. Translationally, their findings suggest that externally-observed behavioral classification of psychiatric disorders may be limited for understanding etiology, supporting the use of neurorobotics models. This study also modeled a network-wide neural process; as a developmental disorder it would be important to simulate and assess the impact of this error mechanism on cognitive development.

### 2.6. Other Robot Models With Implications for Neurological Disorders

Robotic experiments not directly modeling a specific pathology can nevertheless inform pathophysiology. For example, neural networks have been used in cognitive neurorobotics to understand human cognitive functions, such as memory (Shibata Alnajjar et al., 2013) or language (Marocco et al., 2010), and therefore implications for cognitive dysfunction may be inferred.

An early basal ganglia model by Prescott et al. (2006) using the Khepera I robot in an behavioral task of collecting and consuming food modulated by motivations, such as hunger, showed that behavioral disintegration can occur when confronted with multiple high salience alternatives, with implications for the symptomatology, specifically bradykinesia, in PD. Using a similar task, Lewis and Cañamero (2016) used a NAO robot to investigate the interaction between pleasure, perception, and motivation and found that high pleasure improved viability and flexibility in adaptive behavior, with implications for obsessive-compulsive disorder (OCD). Krichmar (2013) used a CarlRoomba robot with a neural network that modeled neurotransmitters in an open-field test to demonstrate that high serotonin increased anxiety whereas high dopamine led to increased curiosity and risk-taking, which is seen in gambling addiction following long term dopaminergic treatment in PD. Conversely, low dopamine led to a more withdrawn behavior, like in PD. In a more complex model, Lones et al. (2016) used a Koala II robot with a hormone-driven epigenetic mechanism that controlled cognitive development and a novel neural network with learning capabilities to interact with environments of different levels of sensorimotor experience. They showed that the sensory-deprived robot performed worse at adaptation and learning, with potential implications for developmental disorders and rehabilitation. Lastly, neurorobotic models have also been used to investigate mental simulations, such as Seepanomwan et al. (2015) who used an iCub robot with a biologically-plausible decision-making mechanism to do a mental rotation task, which may be used to investigate lesions to different parts of the cortex.

## 3. Discussion

**Figure 1:**
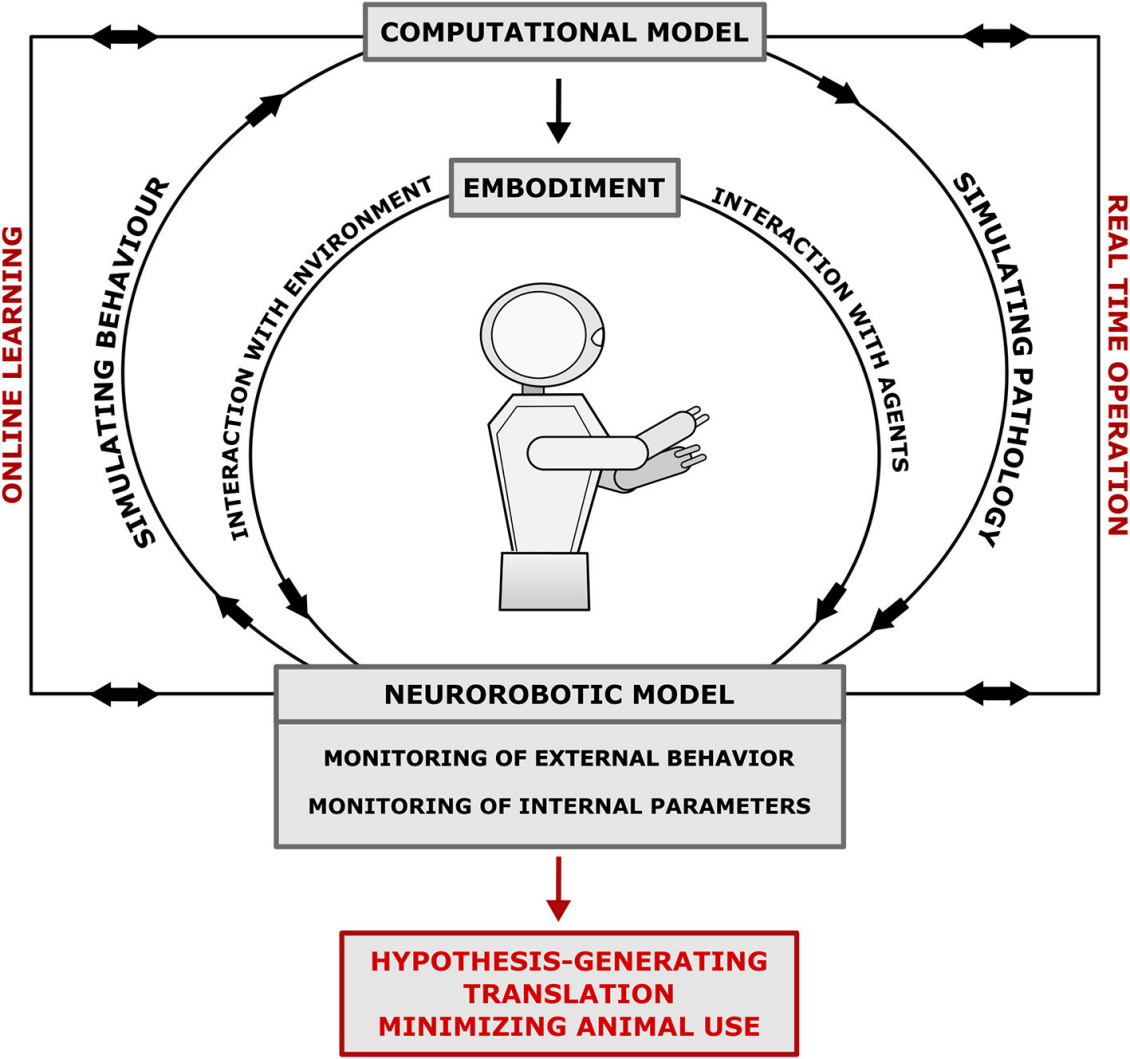
Neurorobotics model workflow of neurological disorders. The EMBODIMENT of the COMPUTATIONAL MODEL in a robot allows the investigation of the link between brain-body-environment with resultant modification of internal parameters via online learning. Additionally, real-time operation allows co-monitoring of internal parameters and external behavior. The model can be designed to simulate pathophysiology or be backwards designed through monitoring the behavior of the robot to mimic symptoms. Monitoring behavior and internal parameters result in hypothesis generation, translation to clinical research, and minimization of animal use. The model is robust, unfatiguable and easily modifiable, which allows for replicable and scalable experiments that efficiently use resources.

The models above demonstrate the utility of robotics in investigating neurological disorders, particularly the ability to monitor both internal parameters and external behavior with the environment. They can generate insights and translational outputs by: manipulating internal parameters; simulating more abstract systemic functions, such as prediction; and monitoring internal states in conjunction with external behavior (Figure 1). Some experiments also demonstrate how these models can be used to guide therapeutics and rehabilitation. Further experiments can be designed, tailored to utilize these advantages by modeling scenarios and measuring parameters beyond the scope of other models. For example, investigating the relationships between neural plasticity, environmental interaction, movement, and cognition in neurological disorders such as PD (Humphries et al., 2019). However, the heterogeneity of robot designs and underlying artificial neural networks employed above highlights a weakness of this modeling platform due to lack of overlap between separate models. Therefore, there is a need to standardize robotic modeling of disorders to improve generalizability of experiments to humans and the interaction between disordered and normal function. Lewis et al. (2019) provide a 12-step 3-stage design framework for creating robot models of neurological disorders and The Neurorobotics Platform of the Human Brain Project aims to simplify the workflow and reduce the level of the required programming skills by allowing researchers to design and run basic experiments in neurorobotics using simulated robots and simulated environments linked to computational neural models that are abstraction of the brain structure and function (Falotico et al., 2017). Some of those simplified models may be suitable for specific analyses but might represent limitations as neural controllers. Nevertheless, increased dialogue between the fields of neurorobotics and computational modeling is required to improve the biological-plausibility of the neural controllers of robots. In conclusion, few neurological disorders have yet been modeled using the neurorobotics approach, each with varying methodologies, however they demonstrate that neurorobotics is a valid modeling platform for a spectrum of different pathologies with unique advantages and potential for translational output.

## Author Contributions

SP wrote the manuscript and the preliminary designs of the table and figure. RM and PV guided the concept and structure of the manuscript. JP created the final figure design. LW created the final table design. All authors reviewed and edited the completed manuscript prior to submission.

## Conflict of Interest

The authors declare that the research was conducted in the absence of any commercial or financial relationships that could be construed as a potential conflict of interest.

## Footnotes

^1^https://www.humanbrainproject.eu/en/brain-simulation/ (accessed January 18, 2021).

## References

[B1] Aguilera M. Barandiaran X. E. Bedia M. G. Seron F. (2015). Self-organized criticality, plasticity and sensorimotor coupling. explorations with a neurorobotic model in a behavioural preference task. PLoS ONE 10:e117465. 10.1371/journal.pone.0117465 25706744 PMC4338039

[B2] Albin R. L. Young A. B. Penney J. B. (1989). The functional anatomy of basal ganglia disorders. Trends Neurosci. 12, 366–375. 10.1016/0166-2236(89)90074-X 2479133

[B3] Allan C. Couceiro M. S. Vargas P. A. (2015). Uncovering emotional memories in robot soccer players, in 2015 24th IEEE International Symposium on Robot and Human Interactive Communication (RO-MAN), 474–479. 10.1109/ROMAN.2015.7333599

[B4] Asada M. Hosoda K. Kuniyoshi Y. Ishiguro H. Yoshikawa Y. Ogino M.. (2009). Cognitive developmental robotics: a survey. IEEE Trans. Auton. Mental Dev. 1, 12–34. 10.1109/TAMD.2009.2021702

[B5] Caligiore D. Mannella F. Baldassarre G. (2019). Different dopaminergic dysfunctions underlying parkinsonian akinesia and tremor. Front. Neurosci. 13:550. 10.3389/fnins.2019.00550 31191237 PMC6549580

[B6] Caligiore D. Tommasino P. Sperati V. Baldassarre G. (2014). Modular and hierarchical brain organization to understand assimilation, accommodation and their relation to autism in reaching tasks: a developmental robotics hypothesis. Adapt. Behav. 22, 304–329. 10.1177/1059712314539710

[B7] Chaminade T. Cheng G. (2009). Social cognitive neuroscience and humanoid robotics. J. Physiol. 103, 286–295. 10.1016/j.jphysparis.2009.08.011 19665550

[B8] Cohen I. (1994). An artificial neural network analogue of learning in autism biological psychiatry. Biol. Psychiatry 36, 5–20. 10.1016/0006-3223(94)90057-4 8080903

[B9] Cohen L. Billard A. (2016). Influence of saliency and social impairments on the development of intention recognition, in Artificial Neural Networks and Machine Learning-ICANN 2016, eds Villa A. E. Masulli P. Pons Rivero A. J. (Cham: Springer International Publishing), 205–213. 10.1007/978-3-319-44778-0_24

[B10] Connolly C. Burns J. Jog M. (2000). A dynamical-systems model for Parkinson's disease. Biol. Cybernet. 83, 47–59. 10.1007/PL00007971 10933237

[B11] Conti D. Di Nuovo S. Cangelosi A. Di Nuovo A. (2016). Lateral specialization in unilateral spatial neglect: a cognitive robotics model. Cogn. Process. 17, 321–328. 10.1007/s10339-016-0761-x 27018020 PMC4933727

[B12] Di Nuovo A. G. Marocco D. Di Nuovo S. Cangelosi A. (2013). Autonomous learning in humanoid robotics through mental imagery. Neural Netw. 41, 147–155. 10.1016/j.neunet.2012.09.019 23122490

[B13] Dovzhenok A. Rubchinsky L. L. (2012). On the origin of tremor in Parkinson's disease. PLoS ONE 7:e41598. 10.1371/journal.pone.0041598 22848541 PMC3407214

[B14] Ericsson A. C. Crim M. J. Franklin C. L. (2013). A brief history of animal modeling. Missouri Med. 110, 201–205. 23829102 PMC3979591

[B15] Falotico E. Vannucci L. Ambrosano A. Albanese U. Ulbrich S. Tieck J. C. V.. (2017). Connecting artificial brains to robots in a comprehensive simulation framework: the neurorobotics platform. Front Neurorobot. 11:2. 10.3389/fnbot.2017.00002 28179882 PMC5263131

[B16] Frank M. (2005). Dynamic dopamine modulation in the basal ganglia: a neurocomputational account of cognitive deficits in medicated and nonmedicated parkinsonism. J. Cogn. Neurosci. 17, 51–72. 10.1162/0898929052880093 15701239

[B17] Humphries M. D. Obeso J. A. Dreyer J. K. (2018). Insights into Parkinson's disease from computational models of the basal ganglia. J. Neurol. Neurosurg. Psychiatry 89, 1181–1188. 10.1136/jnnp-2017-315922 29666208 PMC6124639

[B18] Humphries M. D. Stewart R. D. Gurney K. N. (2006). A physiologically plausible model of action selection and oscillatory activity in the basal ganglia. J. Neurosci. 26, 12921–12942. 10.1523/JNEUROSCI.3486-06.2006 17167083 PMC6674973

[B19] Humphries S. Klooster N. Cardillo E. Weintraub D. Rick J. Chatterjee A. (2019). From action to abstraction: the sensorimotor grounding of metaphor in Parkinson's disease. Cortex 121, 362–384. 10.1016/j.cortex.2019.09.005 31678683 PMC6903422

[B20] Ichimura D. Yamazaki T. (2019). A pathological condition affects motor modules in a bipedal locomotion model. Front. Neurorobot. 13:79. 10.3389/fnbot.2019.00079 31616276 PMC6763684

[B21] Idei H. Murata S. Chen Y. Yamashita Y. Tani J. Ogata T. (2017). Reduced behavioral flexibility by aberrant sensory precision in autism spectrum disorder: a neurorobotics experiment, in 2017 Joint IEEE International Conference on Development and Learning and Epigenetic Robotics (ICDL-EpiRob) (Lisbon), 271–276. 10.1109/DEVLRN.2017.8329817

[B22] Idei H. Murata S. Chen Y. Yamashita Y. Tani J. Ogata T. (2018). A neurorobotics simulation of autistic behavior induced by unusual sensory precision. Comput. Psychiatry 2, 164–182. 10.1162/CPSY_a_00019 30627669 PMC6317752

[B23] Kennedy D. P. Adolphs R. (2012). The social brain in psychiatric and neurological disorders. Trends Cogn. Sci. 16, 559–572. 10.1016/j.tics.2012.09.006 23047070 PMC3606817

[B24] Krichmar J. L. (2013). A neurorobotic platform to test the influence of neuromodulatory signaling on anxious and curious behavior. Front. Neurorobot. 7:1. 10.3389/fnbot.2013.00001 23386829 PMC3564231

[B25] Kumaravelu K. Brocker D. T. Grill W. M. (2016). A biophysical model of the cortex-basal ganglia-thalamus network in the 6-ohda lesioned rat model of Parkinson's disease. J. Comput. Neurosci. 40, 207–229. 10.1007/s10827-016-0593-9 26867734 PMC4975943

[B26] Lanillos P. Oliva D. Philippsen A. Yamashita Y. Nagai Y. Cheng G. (2020). A review on neural network models of schizophrenia and autism spectrum disorder. Neural Netw. 122, 338–363. 10.1016/j.neunet.2019.10.014 31760370

[B27] Lanyon L. J. Denham S. L. (2004). A model of active visual search with object-based attention guiding scan paths. Neural Netw. 17, 873–897. 10.1016/j.neunet.2004.03.012 15288904

[B28] Lawson R. P. Rees G. Friston K. J. (2014). An aberrant precision account of autism. Front. Hum. Neurosci. 8:302. 10.3389/fnhum.2014.00302 24860482 PMC4030191

[B29] Lewis M. Cañamero L. (2019). A robot model of stress-induced compulsive behavior, in 2019 8th International Conference on Affective Computing and Intelligent Interaction (ACII) (Cambridge), 559–565. 10.1109/ACII.2019.8925511

[B30] Lewis M. Cañamero L. (2016). Hedonic quality or reward? A study of basic pleasure in homeostasis and decision making of a motivated autonomous robot. Adapt. Behav. 24, 267–291. 10.1177/1059712316666331 28018120 PMC5152795

[B31] Lewis M. Fineberg N. Cañamero L. (2019). A robot model of oc-spectrum disorders: design framework, implementation, and first experiments. Comput. Psychiatry 3, 40–75. 10.1162/CPSY_a_00025

[B32] Lones J. Lewis M. Cañamero L. (2016). From sensorimotor experiences to cognitive development: investigating the influence of experiential diversity on the development of an epigenetic robot. Front. Robot. AI 3:44. 10.3389/frobt.2016.00044

[B33] Marocco D. Cangelosi A. Fischer K. Belpaeme T. (2010). Grounding action words in the sensorimotor interaction with the world: experiments with a simulated icub humanoid robot. Front. Neurorobot. 4:7. 10.3389/fnbot.2010.00007 20725503 PMC2901088

[B34] McGregor M. M. Nelson A. B. (2019). Circuit mechanisms of Parkinson's disease. Neuron 101, 1042–1056. 10.1016/j.neuron.2019.03.004 30897356

[B35] Mozer M. Halligan P. Marshall J. (1997). The end of the line for a brain-damaged model of unilateral neglect. J. Cogn. Neurosci. 9, 171–190. 10.1162/jocn.1997.9.2.171 23962010

[B36] Pio-Lopez L. Nizard A. Friston K. Pezzulo G. (2016). Active inference and robot control: a case study. J. R. Soc. Interface 13. 10.1098/rsif.2016.0616 27683002 PMC5046960

[B37] Poewe W. Seppi K. Tanner C. M. Halliday G. M. Brundin P. Volkmann J.. (2017). Parkinson disease. Nat. Rev. Dis. Primers 3, 1–21. 10.1038/nrdp.2017.13 28332488

[B38] Prescott T. J. Montes González F. M. Gurney K. Humphries M. D. Redgrave P. (2006). A robot model of the basal ganglia: behavior and intrinsic processing. Neural Netw. 19, 31–61. 10.1016/j.neunet.2005.06.049 16153803

[B39] Richardson R. M. (2017). Global brain initiatives. Neurosurgery 80, N21–N22. 10.1093/neuros/nyx118 28586494

[B40] Russell W. M. S. Burch R. L. (1960). The principles of humane experimental technique. Med. J. Austr. 1:500. 10.5694/j.1326-5377.1960.tb73127.x

[B41] Seepanomwan K. Caligiore D. Cangelosi A. Baldassarre G. (2015). Generalisation, decision making, and embodiment effects in mental rotation: a neurorobotic architecture tested with a humanoid robot. Neural Netw. 72, 31–47. 10.1016/j.neunet.2015.09.010 26604095

[B42] Shibata Alnajjar F. Yamashita Y. Tani J. (2013). The hierarchical and functional connectivity of higher-order cognitive mechanisms: neurorobotic model to investigate the stability and flexibility of working memory. Front. Neurorobot. 7:2. 10.3389/fnbot.2013.00002 23423881 PMC3575058

[B43] Smith L. Gasser M. (2005). The development of embodied cognition: six lessons from babies. Artif. Life 11, 13–29. 10.1162/1064546053278973 15811218

[B44] van der Worp H. B. Howells D. W. Sena E. S. Porritt M. J. Rewell S. O'Collins V.. (2010). Can animal models of disease reliably inform human studies? PLoS Med. 7:e1000245. 10.1371/journal.pmed.1000245 20361020 PMC2846855

[B45] WHO (1993). The ICD-10 Classification of Mental and Behavioral Disorders: Diagnostic Criteria for Research. Geneva: World Health Organization (WHO).

[B46] Yamashita Y. Tani J. (2012). Spontaneous prediction error generation in schizophrenia. PLoS ONE 7:e37843. 10.1371/journal.pone.0037843 22666398 PMC3364276

[B47] Yiping W. Qingwei C. Weili H. (2010). Behavior selection mechanism of two typical brain movement disorders: comparative study using robot, in 2010 International Conference on Digital Manufacturing Automation, Vol. 1 (Changcha), 319–323. 10.1109/ICDMA.2010.458

